# Brains for dementia research at 18: Commitment to impact the value of human *post‐mortem* tissue

**DOI:** 10.1002/alz.71956

**Published:** 2026-10-26

**Authors:** Keeley J. Brookes, Nicola Barnett, Johanna S. Jackson, Emma J Kidd, Debra J. Lett, Jillian Madine, Ben Middlehurst, Laura E. Palmer, Laura Parkkinen, Andrew C. Robinson, Rebecca Sims, Lindsey Sinclair, Alexandra Stolzing, Philip Tinkler, Claire Troakes, Lauren Walker, The BDR Collaborative, Alan Thomas, Patrick G. Kehoe

**Affiliations:** ^1^ Biosciences Nottingham Trent University Nottingham UK; ^2^ Faculty of Medical Sciences Newcastle University Newcastle UK; ^3^ UK Dementia Research Institute Imperial College London London UK; ^4^ Department of Brain Sciences Imperial College London London UK; ^5^ School of Pharmacy and Pharmaceutical Sciences Cardiff University Cardiff UK; ^6^ Translational and Clinical Research Institute Newcastle University Newcastle upon Tyne UK; ^7^ Institute of Systems, Integrative and Molecular Biology University of Liverpool Liverpool UK; ^8^ South West Dementia Brain Bank University of Bristol Bristol UK; ^9^ The Oxford Brain Bank, Nuffield Department of Clinical Neurosciences University of Oxford Oxford UK; ^10^ Faculty of Biology, Medicine and Health, School of Biological Sciences, Division of Neuroscience The University of Manchester Manchester UK; ^11^ Geoffrey Jefferson Brain Research Centre The University of Manchester Manchester UK; ^12^ Division of Psychological Medicine and Clinical Neuroscience, School of Medicine Cardiff University Cardiff UK; ^13^ Department of Psychiatry University of Southampton Southampton UK; ^14^ Centre for Biological Engineering, Wolfson School of Mechanical, Electrical and Manufacturing Engineering Loughborough University Loughborough UK; ^15^ London Neurodegenerative Diseases Brain Bank, Institute of Psychiatry, Psychology and Neuroscience King's College London London UK; ^16^ School of Health & Life Sciences Teesside University Middlesbrough UK; ^17^ Biomedical Research Centre Newcastle University Newcastle UK; ^18^ School of Psychology and Neuroscience, Faculty of Health and Life Sciences University of Bristol Bristol UK

**Keywords:** BDR, dementia, longitudinal cohort, *post‐mortem* human brain tissue

## Abstract

The Brains for Dementia Research (BDR) project was established to provide high quality, neuropathologically characterized *post‐mortem* brain tissue from a cohort of participants longitudinally assessed for outcomes that are relevant for research in relation to dementia. From its origins in 2008, over 3200 individuals with and without dementia consented to participate in the project and to donate their brains after death. Now, 18 years later over half of these pledges have been collected. Here we celebrate the BDR project and provide an update on its contributions to the field of dementia research thus far, and the future promise of maximizing benefit from this endeavor with the conception of the “BDR Collaborative”. The BDR Collaborative seeks to align and intersect data generated on this cohort and to accelerate advances in our understanding of neurodegeneration.

## Highlights

- Longitudinal highly phenotyped cohort
- Matched *post‐mortem* brain tissue and blood
- Multi'omics data
- Collaborative Project

## 1 OVERVIEW OF THE BRAINS FOR DEMENTIA RESEARCH PROJECT

Recruitment to the Brains for Dementia Research (BDR) project started in 2008. Its aim was to create a collection of *post‐mortem* brain tissue across the United Kingdom for dementia research, assessed in a standardized way *pre mortem* using dementia‐relevant assessment criteria and similarly using standardized protocols for neuropathological assessment *post‐mortem*. The cohort comprised participants with dementia as well as older individuals who were cognitively normal. There was then and continues to be a pressing need in the dementia research field for well‐characterized brain tissue accompanied by extensive, serially acquired, clinically relevant data to aid in the understanding of brain pathology related to dementia.^1^, ^2^Furthermore, the project design involved patient and carer representation in both the set‐up and management of the project. Given the strong desire of people with direct experience of dementia to donate their own, or a family members’ brain to aid in the research of this disease, coupled with the longevity of the project, it was vital to have their input to ensure high levels of understanding, communication, and retention of participants.^3^The value of this is highlighted by a testimonial from the family of a BDR participant that stated “Experiencing the sadness of dementia and needing to do something about it made it an easy decision to donate Dad's brain to BDR. In retrospect it has been comforting to know that some small part of Dad has been able to assist in the important research for dementia which we hope will be of benefit to others.”

BDR set out with the following core aims which endure today:

- Raise public awareness of brain donation for dementia research.
- Increase the number of brain tissue donations to allow comparisons to be made between “normal” aging and different types of dementia.
- Standardize tissue processing and assessment protocols to minimize heterogeneity between brain banks.
- Provide the highest quality tissue to support research with *pre‐mortem* longitudinal clinically relevant, cognitive and behavioral data.
- Increase access to tissue for researchers via a searchable online database.
- Increase the number of research studies using validated human *post‐mortem* material.

BDR was initially funded through the Alzheimer's Brain Bank UK initiative (ABBUK) which was funded collaboratively through a joint venture established by the Alzheimer's Society and Alzheimer's Research UK (ARUK). BDR has been directed and managed by a Coordinating Centre and comprises of a network of five brain banks based at the Universities of King's College London, Oxford, Bristol, Newcastle and Manchester, alongside an additional participant recruitment center at Cardiff. The study was originally advertised in national and local press in radio and TV coverage and through charity newsletters, magazines, and organizations (e.g. University of the Third Age) aimed at an older demographic. Potential donors already registered with the brain banks were also invited to the join the program. All participants were self‐referring (whether cognitively healthy or otherwise) or referred by family members acting as a consultee where the participant lacked capacity.

BDR has been funded in multiple phases BDR1: 2008–2013, BDR2: 2013–2018, BDR3: 2018–2021, and is now in BDR4 running until spring 2028. Currently work is underway with a new Lead Centre to develop a new strategic plan for BDR5.

The donated brain tissue is held within UK Brain Bank Network (UKBBN) database. The UKBBN was originally funded by the Medical Research Council until April 2023. At this time, the UKBBN database was adopted by the funders of BDR to continue its purpose of providing a coordinated searchable national database of brain tissue and relevant information for researchers to use across the globe.

## 2 THE BDR COHORT: SCALE, STRUCTURE, AND ACCESSIBILITY

BDR closed recruitment in 2018 when a total of 3219 participants had consented to take part (Table S1). Of these almost 95% (*n* = 3095) completed the baseline assessment. The cohort is predominantly of white ethnicity (98.9%), 59.9% female, and has a median of 13 years of education.^1^As of July 2026; 1559 (50% of baseline assessments) participants have died with 1355 brains (86.9%) successfully donated for research purposes. At the time of writing, 1072 donations are still anticipated. To date, the retention rate exceeds the average retention rate for longitudinal studies reported in a meta‐analysis by Teague and colleagues^4^which found a mean retention rate of 73.9% (SD = 20.1%) across 143 longitudinal studies ranging from 6 months to 30 years with an average of 4.1 years (SD = 5.1 years). A similar endeavor (Longitudinal Aging Study Amsterdam) running over 17 years, with 3177 participants at recruitment had a reported retention rate of 32%.^4^

Demographic data and any relevant clinical findings, medication history, cause of death, are stored for each brain donation on the UKBBN database. In addition are notes on the availability of BDR assessment data alongside the neuropathological findings from *post‐mortem* examination, and the availability of different types of samples for study (frozen brain tissue, formalin‐fixed paraffin‐embedded brain tissue, blood samples, and induced pluripotent stem cells). Where available, genetic information such as apolipoprotein E (*APOE)* genotype is also stored along with other polymorphisms and mutations associated with neurological disease. UKBBN database has been progressively refined to standardize the recording of data and to improve ease of use and search functionality.

Data is uploaded by participating brain banks, all of whom have their own research ethics approval for the collection and storage of brain tissue samples and accompanying patient data, and function as National Health Service (NHS) Research Ethics Committee approved Research Tissue Banks.

Access to the database is open to researchers across the globe following registration, and it allows selection of donors from whom tissue samples can be requested for scientific investigation employing a variety of user specifications, including via cohort. Tissue requests are made directly to the relevant brain bank and are subject to their standard review processes and cost‐recovery tariffs. Project proposals involving data, cell lines or *pre‐mortem* bloods are reviewed by the BDR Unified Access Committee, which is composed of scientific expertise as well as public and funding body representatives, requiring approvals from all three stakeholders to proceed.

BDR adopts a standardized *post‐mortem* code of conduct developed by the BrainNet Europe (BNE) consortium^5^ and blocking format which is comparable to the US Alzheimer's Disease Research Centers (ADRC), funded via NIA‐AA.^6^ At least 15 brain areas are embedded as formalin fixed blocks utilizing one side of the hemisphere (Table S2), while this remains the standard preservation technique for *post‐mortem* brain tissue, the BDR banks continually work with tissue users to adapt procedures for the most current experimental techniques. The other hemisphere is frozen, with a narrative description of the regional pathology within the brain following standardized scoring of pathology^7^(Table S3) block and coronal slices prior to freezing. The brain banking alongside a unified dementia‐relevant assessment regimen across all centers, creates a robust and reproducible method of diagnostic reporting.

The advantage of BDR being both a longitudinal study and a provider of well‐characterized *post‐mortem* brain tissue is that the enrolled participants are continuously assessed throughout later life. The full BDR Interview Schedule includes age, general health, body measurements, cognitive tests (Clinical Dementia Rating [CDR], Montreal Cognitive Asessment [MoCA], Mini‐Mental State Examinatin [MMSE]), physical health (activity profiles, gait speed, grip strength), diet (14‐item Mediterranean Diet Adherence Screener [MEDAS]), alcohol consumption, and smoking frequency. This enables the intersection of epidemiology and biological data to make meaningful links between lifestyle and pathology. Many lifestyle factors are now recognized as modifiable risks for dementia.^8^, ^9^

Alongside the Interview Schedule, blood samples are now collected at regular intervals, providing whole blood, serum, plasma and PAXgene (RNA) samples for investigation. These samples are highly valuable for exploring disease progression and will support the search for blood biomarkers to identify individuals at risk of dementia, earlier, before symptoms appear and potentially irreversible damage occurs. BDR currently hold 959 venepunctures taken from 513 of the participants; at the time of writing 126 of these blood samples have matching *post‐mortem* brain tissue.

In 2019, another strand to the BDR project was launched when BDR participants were approached to give their consent to their tissue being used to create fibroblasts and stem cells. To date, 52 cell lines have been generated and are being uploaded to the international stem cell registry at hPSCreg, whereas 164 fibroblasts lines are available across phenotypic groups (Table S4).

Each iPSC clone is tested for the ability to maintain pluripotency and for the capacity to differentiate into the three germ cell layers. Clones are then tested using the Neurobooster array^10^to provide genotype information and compare with tissue DNA. Cell lines are screened for human immunodeficiency virus (HIV) 1 and 2, hepatitis (HEP) B and C, severe acute respiratory syndrome coronavirus 2 (SARSCoV2), and mycoplasma. Consequently, the cells have neuropathological verification of disease or control status from a range of diagnostic categories which include Alzheimer's disease (AD), dementia with Lewy bodies (DLB), mixed AD/DLB, vascular dementia (VaD), frontotemporal lobar dementia (FTLD), primary age‐related tauopathy (PART), multiple system atrophy (MSA), progressive supranuclear palsy (PSP) as well as normal controls. All cases can be found on UKBBN and the availability of such diagnosed cell lines together with associated data, brain tissue and other samples, provides researchers with a wealth of resources over and above those typically offered by aging cohorts.

In 2014 funding was granted for a systematic and concerted effort toward providing genetic data on the entire BDR cohort as tissue was collected. The genetic data from the initial analysis^11^ conducted on the Neurochip platform covering 466,947 variants^12^released in 2021, is freely available for 772 samples from *post‐mortem* tissue and 400 of the living participants via the Dementias Platform UK (DPUK). The DNA bank now holds 1442 DNA samples from the cohort (*post‐mortem* and living) with a new iteration of genetic data. The new Neurobooster array data captures 1.825 million genetic variants from the existing Global Diversity Array with an additional 95,273 variants associated with more than 70 neurological conditions or traits. It features approximately 10,000 tagging variants facilitating imputation for up to 15 million genetic variants across diverse populations, allowing for even greater exploration of the genome^10^or enabling integration with other cohorts or providing an independent replication dataset, which is needed due to large scale heterogeneity within larger datasets.^13^The data is now freely available via request to the BDR Lead Centre (manuscript under review).

Alongside the genetic data, DNA methylation profiles have been generated on just under half of the BDR *post‐mortem* cohort. Over 850,000 methylation sites across the human genome are available for interrogation via the Illumina EPIC DNA Methylation Array^14^ conducted on 632 bulk tissues from the prefrontal cortex and occipital cortex of BDR participants.^15^

## 3 MEASURING INFLUENCE: HOW RESEARCHERS USE THE BDR

While living model organisms or engineered organoids allow for controlled prospective studies of disease mechanisms, the primary strength of *post‐mortem* brain tissue lies in the ability to investigate real‐world human specimens, where multiple aspects of neurodegeneration can be considered.^16^, ^17^Human *post‐mortem* tissue has been instrumental in supporting and validating model system investigations; providing pilot data for new techniques and aligning pathology with phenotypes and genetics/genomics.^18^

Already, BDR has supported a range of studies that span the full breadth of neurodegeneration research. As of July 2026, BDR has been cited in over 500 scientific publications, providing a steady impact to the literature from 2014 onward (Figure 1) Tissue samples from BDR have been selected based on specific diagnoses, neuropathology, genetic, or demographic parameters, leading to key observations that have significantly contributed to the understanding of dementia.

**FIGURE 1:**
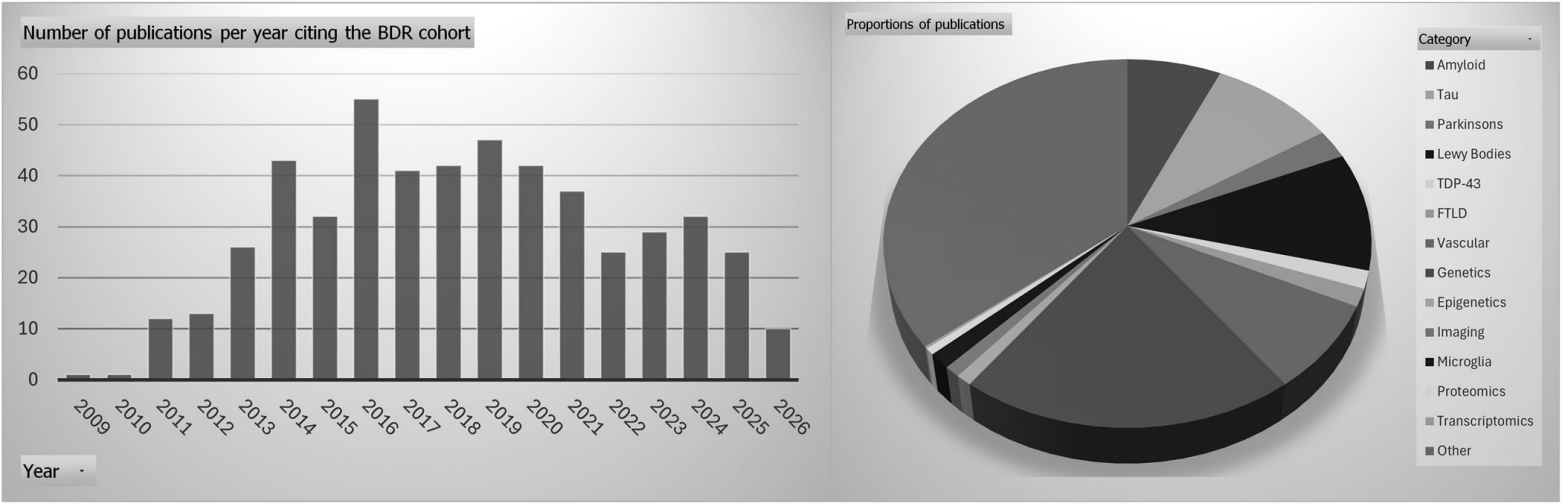
Summary of publications citing use of the BDR cohort. A total of 513 publications utilizing BDR have generated since 2009 to‐date. The topics are broad‐ranging and cover all dementia types, key pathologies, and data types. BDR, Brains for Dementia Research.

The UKBBN database has been highly successful in facilitating research on donated brain tissue, with 1562 registered users and over 400 logins per month (based on the last 12 months). This usage as led to more than 300 tissue requests each year and the provision of over 75,000 samples annually to research groups across the United Kingdom and internationally.

DPUK, is a secure data portal for housing much of the BDR data as well as other cohorts. To date (July 2026), there have been 44 successful applications to access BDR data from the United Kingdom and beyond, and BDR is consistently in the top two DPUK most requested cohorts. The project is used as an example cohort at the DPUK training academies, introducing early career researchers to what BDR has to offer and in 2020, when DPUK and partners launched the Alzheimer's Disease Data Initiative (ADDI), known as the AD Workbench, BDR were keen to be onboard. This initiative was established with a vision to create an innovative data platform enabling academics, industry, and others to share their data to further our understanding of dementias, and BDR are now in receipt of applications to access data via this platform. The BDR link with DPUK goes beyond data sharing, as many of the BDR participants were offered the opportunity to increase data capture by joining the Great Minds Study and an opt‐out register of volunteers termed the Clinical Studies Register, thereby facilitating targeted recontact of living BDR participants. Koychev *et al*.,^19^stress the added value which comes from recruiting participants with well‐characterized data to new studies as they become available. To‐date, failure in clinical trials of dementia drugs may largely be due to a lack of specificity and they conclude that stratified recruitment into early phase experimental medicine studies is key to reducing risks (both medical and financial) and to increasing investment in neuroscience research and development. The DPUK recontact platform therefore provides an opportunity for the BDR cohort to accelerate research into novel dementia treatments, through the linkage of highly characterized individuals with experts in the dementia research field.

### 3.1 BDR's role in shaping best practice in brain banking and neuropathological definitions

The collection of brain tissue for research alongside serial assessments and cognitive data present considerable challenges. Expanding cohorts require increasing resources to repeated assessment data, as the cohort expands often over larger geographical locations. Maintaining acceptable assessment intervals is difficult especially when monitoring the conversion to dementia. Likewise healthy controls live longer and have more active lifestyles from time of consent to death and present availability challenges. Therefore, enhancing the capacity to conduct assessments and manage cohorts more effectively is instrumental for ensuring scientifically informative brain donation programmes.^20^Although face‐to‐face neuropsychiatric interviews, are generally considered the “gold standard”, a BDR pilot study demonstrated the feasibility of telephone assessments with healthy volunteers, reducing the assessment burden without jeopardizing participant retention.^20^This included the Telephone Interview Cognitive Status (TICS) assessment^21^which has been found to be an effective screening tool for cognitive impairment and early identification of individuals at risk of developing dementia.^22^Telephone assessments were crucial during the coronavirus disease 201 (COVID‐19) pandemic allowing data collection to be continued. However, it is important to note that the selection of participants for telephone assessments is paramount, and it may not be suitable for all participants due to age‐related hearing loss nor used in isolation without some in‐person assessments.

Neuropathological examination has revealed that more than a third of cases studied were dis‐concordant with the clinical diagnosis, and that mixed pathology is more prominent than previously recognised.^23^A further study including BDR samples observed AD associated pathology and cerebral amyloid angiopathy in those defined as cognitively healthy individuals at the time of death^24^possibly indicating death occurred before the onset of observable symptoms or a cognitively resilient sub‐population. With up to 25% of clinically normal BDR participants found to have intermediate phosphorylated‐tau (pTau) pathology, this indicates the strength of investigating neuropathological features of disease pathology over clinical diagnoses.^23^, ^25^Further to this the presence of neurofibrillary tangles without associated amyloid pathology is noted in the BDR cohort. PART has been identified in cognitively impaired individuals as well as those displaying normal cognitive profiles and is associated with the presence of the APOE ε2 isoform indicated by a study conducted on BDR samples.^26^

Current staging criteria for the assessment of α‐synuclein in Lewy body disease (LBD) are based on distribution of Lewy bodies and Lewy neurites throughout the brain. However, as individuals with LBD pathologies display a high degree of heterogeneity, not all cases, particularly incidental cases, are classified by these criteria. To address this, Attems *et al.*,^27^used BDR cases to update the current criteria. In addition to brainstem, limbic and neocortical stages, amygdala‐predominant and olfactory‐only stages were added. This revision allowed 100% of cases to be classified reliably with high reproducibility and these criteria are now incorporated into BDR neuropathology reports.

Aggregated phosphorylated Tar DNA‐binding Protein 43 (TDP‐43) pathology is typically associated with FTLD; however, studies utilizing the BDR cohort demonstrated the presence of TDP‐43 in AD, DLB, mixed AD/DLB cases and in a significant number of older controls.^28^This indicates that TDP‐43 may be an important yet overlooked pathological hallmark to assess in *post‐mortem* brain studies.

In LBD, additional pathologies not typically associated i.e. (pTau, amyloid β, and TDP‐43) are frequently observed at *post‐mortem* examination and are associated with a poorer clinical outcome for patients. BDR brain tissue has been instrumental in identifying mixed pathologies contributing toward clinical and neuropathological phenotypes.^29^, ^30^Howlett and colleagues^29^ demonstrated a combined pathology score rather than a single pathology, was predictive of the development of dementia. More recently, Walker and colleagues^30^highlighted that the prevalence and distribution of cerebral amyloid angiopathy differs between AD and LBD cases, which may have implications for amyloid‐based therapies.

Until 2016, there were no widely accepted protocols for the assessment of vascular pathology in dementia. To address this, UK neuropathologists, including those at BDR centers collaborated to develop the Vascular Cognitive Impairment Neuropathology Guidelines (VCING). This framework uses a combination of three main neuropathological features to assign a low, intermediate or high likelihood that cerebrovascular disease contributed to the observed cognitive impairment. The incorporation of VCING enables a comprehensive neuropathological profile for each BDR participant underscoring their dementia presentation.^31^

### 3.2 BDR's role in dispelling myths in *post‐mortem* tissue banking

BDR provides a range of sample types including frozen and formalin‐fixed paraffin‐embedded (FFPE) *post‐mortem* brain tissue depending on the needs of the project. FFPE processing allows for long‐term storage at ambient temperature but is often underutilized due to the assumption that this preservation method is suboptimal for certain types of analysis. However, studies from the Manchester Brain Bank utilizing BDR samples have highlighted that key dementia relevant analyses remain feasible using these fixed tissue samples.^32^, ^33^

Studies highlighting the presence of mixed pathologies within various clinical diagnoses led to the Manchester Brain Bank investigating the ability to identify TDP‐43 in FFPE tissues.^33^Despite regional, and a general weaker immunoreaction signal, phosphorylated TDP‐43 (S409/410) was detected in samples even after extended periods of fixation time (up to 21 years). Alongside an indication of brain tissue being TDP‐43 positive, annotations for the presence of Limbic‐predominant Age‐related TDP‐43 Encephalopathy (LATE) are also included in BDR information.

FFPE preserved tissue also presents challenges for DNA extraction and genotyping, due to DNA fragmentation and cross‐linking caused by the fixation process. Studies assessing the genotyping of the *APOE* isoform single‐nucleotide polymorphisms (SNPs) (rs429358 & rs7412) in cerebellar FFPE tissue preserved for up to 19 years showed that restriction fragment length polymorphism (RFLP) polymerase chain reaction (PCR) could successfully determine genotypes of tissues embedded in paraffin for less than 4 years.^32^This approach has ensured that *APOE* genotypes are available even for those samples without frozen tissue available and enabling potential inclusion in whole genome analyses.

Similarly, Robinson *et al.* undertook a collaborative study^34^ as part of the BDR Neuropathology study group on over 500 frozen brain samples to address the common perception that long *post‐mortem* delays (PMDs) compromise brain quality, and therefore the reliability of scientific data. Although observed RNA Integrity Number (RIN) values decline with decreasing tissue pH, the RIN values nor pH significantly correlated with PMD. Consequently, BDR brain banks no longer measure RIN routinely and rely on tissue pH as a more helpful indicator of tissue integrity and quality than PMD alone.

### 3.3 BDR's contribution to genomic aetiology

In addition to the routine *APOE* genotyping, samples from the BDR cohort have contributed to genetic studies for over 15 years with samples selected by diagnosis or neuropathology from the brain banks, and genetic analysis derived from various genotyping platforms and whole exome and genome sequencing efforts. The first study, published in 2010, combined BDR samples with other cohorts genotyped on the Affymetrix 6.0 array identifying SNP associations with the *CLU, CR1*, and *PICALM* genes,^35^now established gene candidates for AD.

Although relatively small, this dataset is powerful for comparing between neuropathological phenotypes^36^generating data‐driven hypotheses on underlying pathologies and overlap between dementias and neurodegenerative disorders. BDR genetic data has been used to explore associations within the X‐chromosome and neuropathology measures,^37^, ^38^ as well as contributions in the development of polygenic risk score analyses.^39^, ^40^, ^41^Exome‐sequencing on sub‐sets of the cohort has allowed the exploration of rare variants, suggesting that variants in genes such as amyloid precursor protein (*APP*), presenilin (*PSEN1, PSEN2*), and microtubule‐associated protein tau (*MAPT)* may occur in up to 10% of AD cases and 6% of controls.^42^, ^43^

In addition to being utilized for genome‐wide analysis,^11^, ^44^samples from the BDR project have also been genotyped in targeted candidate gene studies including but not limited to *PLD3, ABCA7, ITPR1, LRKK2, GBA TFAM*, *OAS1, and PLAUR*.^45^, ^46^, ^47^, ^48^, ^49^, ^50^, ^51^, ^52^

The breadth of BDR recruitment captures rarer neurodegenerative and dementia‐related phenotypes, increasing the cohort's versatility. BDR samples have been used in genome‐wide associaton studies (GWAS) studies for several disorders including progressive supranuclear palsy^53^; FTLD,^54^, ^55^tauopathies,^56^ and vascular pathology.^57^Capitalising on the extensive neuropathological data has also enabled the correlation of genetic risk factors between dementias.^58^

Studies on BDR samples have investigated the C9orf72 polymorphism, associated with FTLD and amyotrophic lateral sclerosis (ALS), and with TDP‐43 pathology.^59^Given the widespread observations of TDP‐43 presence within AD and LBD samples in BDR—there is perhaps a rationale for genotyping the entire cohort for the hexanucleotide expansion, quantifying the resulting dipeptide repeat proteins to assess their role of TDP‐43 pathology in all dementias.^60^

As with the genetic data, BDR methylation data has been utilized in multiple ways including the development of the “DNA methylation age clock”, by providing independent replication of a 347 methylation sites algorithm to predict cortex age in humans, potentially leading to interesting implications for aging mechanisms in neurodegeneration.^15^BDR data has been combined with other cohorts in large meta‐analyses of methylation in relation to neuropathology data,^61^highlighting differential methylation in the *ANK1*, *ABR, SPG7, WDR81, DUSP27*, and *RHBDF2* genes. In addition, the data has been utilized to investigate less frequent neurodegenerative diseases such as multi‐system atrophy^62^ and Lewy body pathology.^63^Intriguingly, comparison of bulk tissue data with neuronal and non‐neuronal specific nuclei methylation profiles, suggest that the majority of differential methylation observed to be associated with AD neuropathology is driven by non‐neuronal cell types.^64^

### 3.4 BDR's advances in facilitating longitudinal analyses to understand disease progression

Findings from BDR support research suggesting modifiable risks could be crucial in slowing or prevention of dementia. Observations of BDR participants indicate that individuals from the most deprived areas of the United Kingdom show significantly greater severity of dementia‐related neuropathology. This risk remaining even when controlling for other factors, including APOE isoforms, co‐morbidities, biological sex, age, and alcohol and tobacco use.^65^

Likewise, Katanga *et al*.,^66^provided support for the association of *ante‐mortem* hearing loss, with the presence and density of neuritic plaques in participants from BDR, although neither non‐specific amyloid deposition nor neurofibrillary tangles were found to be associated.

Rodriguez *et al*.,^67^utilised matched *post‐mortem* brain and plasma samples to investigate whether plasma pTau181 levels could predict neuropathological confirmation of AD and discriminate AD from non‐AD dementia pathologies. They observed that plasma pTau181 levels correlated more strongly with AD neuropathology compared to clinical diagnosis, discriminating AD from non‐AD pathology with > 95% accuracy 8 years prior to death. Similar studies are underway, and the availability of matched blood and *post‐mortem* brain samples strengthens the evidence for plasma pTau181 and additional blood‐based biomarkers for clinical trials and early diagnoses.

### 3.5 BDR tissue contributions to the molecular and cellular basis of neurodegeneration

The wide variety of studies to which BDR has contributed has advanced knowledge in multiple areas of research and are far too numerous to detail fully in this showcase, however some examples are highlighted here. The iTRAQ proteomics platform applied to a small number of BDR samples identified changes in protein levels in vascular dementia, suggesting that hypometabolism, vascular insufficiency, and compromised ion channels activity and inflammation contribute to the pathology.^68^, ^69^While changes in synaptic proteins are observed in AD, DLB, and Parkinson's disease (PD) with dementia, combinations of these proteins could discriminate between these diagnoses and controls with a minimum 73.7% sensitivity with 73.9% specificity.^70^Parallel BDR investigations of the effect of *APOE* genotype on synaptic proteins, observed that APOE ε2 carriers had higher levels of post‐synaptic proteins, particularly drebrin encoded by the *DBN1* gene, which has been shown to have a protective effect against AD in mouse studies.^71^, ^72^

Increased microglia activation associated with dementia phenotypes has been observed in several model organisms for exmaple^73^ and importantly confirmed in human *post‐mortem* tissue. Elevated CD68 positive microglial have been observed in both AD and FTLD compared to controls, across common areas of the brain and distinct regions between pathologies.^74^, ^75^Conversely this increase in microglia activation was not seen in DLB.^76^

Studies on BDR frontal cortex tissue suggest increased expression of genes characteristic of a senescence‐associated pattern, accompanied with elevations of inflammatory markers interleukin (IL) ‐1β and IL‐6.^77^Subsequent mechanistic studies in mice supported this, showing that interrupting microglia proliferation prevented the senescence phenotype, suggesting that early proliferation of activated microglia is followed by replication senescence, putting the microglial on a disease‐associated trajectory driving neuroinflammation.^77^

Given the association of microglia with neuroinflammation the NLRP3 inflammasome is a key candidate for exploration. However, evidence from BDR studies suggest no changes NLRP3 components are observed at either the RNA or protein level.^78^A later studies looking at an upstream regulator of the NLRP3 inflammasome, *KCNK13* was observed to be upregulated with corresponding decreases in DNA methylation of the gene in *post‐mortem* tissue from AD participants of BDR.^79^

## 4 POSITIONING THE BDR WITHIN THE WIDER RESEARCH LANDSCAPE

The BDR project now stands firmly among other longitudinal projects for aging and dementia, both in the United Kingdom and internationally offering a resource for replication and cross‐cohort validation.

Some of the largest studies include two conducted in the United States: the Religious Orders Study/Memory Aging Project (ROSMAP) and Baltimore Longitudinal Study of Aging (BLSA). BLSA, the longest running study, started in 1958^80^ and is still actively recruiting participants. ROSMAP, a recruitment of nuns, priests and monks across the United States aged over 65 years has reached 3414 participants, with blood collected from 94.3% of the cohort and over 1506 *post‐mortem* brain donated and autopsied.^81^Like BDR, both cohorts’ follow‐up their participants with a series of interview schedules and cognitive testing alongside consent for brain donation after death. Both studies have contributed to numerous research papers and have generated an array of multi'omics from the *post‐mortem* tissue, though the overlap of matched datasets across samples is less than 500.^82^, ^83^, ^84^, ^85^

In the United Kingdom, the Cognitive Function and Aging Study (CFAS), recruits participants from Cambridge, Nottingham, and Newcastle, with a complementary study running in Wales. Started in the 1980's, its aim was to recruit over 18,000 participants and is currently in phase II with 7796 participants over 65 years old, taking part in interviews and cognitive testing every 2 years,^86^ again with consent for brain donations being made; at least 300 tissue samples are now available for study.^87^

## 5 FROM EXPERIENCE TO INNOVATION: NEXT STEPS FOR THE BDR

What began as a project focused on facilitating brain donation has evolved into a far‐reaching initiative that has advanced research, fostered collaboration, and strengthened engagement between researchers and the public. Despite the challenges faced by human tissue banking, such as donor retention, sustained funding over decades, continuity and uniformity of protocols, and consistent data collection, cohorts such as BDR provide an essential resource for researchers and a beacon of hope for those living with dementia.

As the project has evolved, so too has the understanding of what is required to deliver a successful and sustainable research initiative. With hindsight, several lessons have emerged that may be relevant not only to future brain donation programmers, but also to the design and delivery of BDR moving forward.

The non‐selective nature of the cohort ensured a wide variety of dementias and cognitively normal individuals are captured; however, such studies have been shown to be subject to demographic bias.^88^It is recognized that participants who volunteer are more likely to have certain characteristics and/or motivations than those who don't, for example, they may be more educated on health concerns. Therefore, self‐selection may lead to a under representation of certain demographic groups. As recruitment to this study is now closed, little can be done to enhance the cohort in terms of demographic diversity, but it yields a clear message that efforts should be undertaken to make sure any study population is representative of different demographic groups. While education efforts of BDR have been directed at its participants and their families (quarterly newsletters and annual public engagement events), perhaps these need to extend into more “hardly reached, seldom‐heard” community groups to address this, providing avenues for a wider diversity if recruitment resumes in the future.

Perhaps an obvious missed opportunity was the routine collection of blood samples at enrolment. This could have provided genetic information allowing stratification of the cohort and the following of aging trajectories, neuropathology, and other outcomes. Similarly, regular blood sampling over‐time would have allowed the evaluation of emerging blood biomarkers, aligned with detailed dementia‐relevant assessments and eventual neuropathological data. The inclusion of addition measures, such as magnetic resonance imaging (MRI), positron emission tomography (PET) and *pre‐mortem* cerebrospinal fluid (CSF) collection would have further enriched the dataset, providing opportunities to examine neuroimaging and fluid biomarkers alongside longitudinal and neuropathological data. However, the costs and logistical challenges for implementing such additions at this scale are largely prohibited within available funding frameworks. As has been the case for the generation of omics datasets, acquisition of these data has relied on external collaborators securing dedicated funding to support their collection and analysis.

The efforts of the BDR project have emphasized the importance of neuropathological investigation alongside dementia‐relevant assessments to inform robust classification of samples for scientific research. The added benefit of genetic and ‘omics data being generated on the entire cohort allow new analyses to be incorporated into investigations and new avenues for data driven hypotheses.^89^, ^90^, ^91^

Ongoing DNA banking, and the generation of comprehensive ‘omics data is paramount for enabling machine‐learning algorithms to align, intersect, and cluster ‘omics data with neuropathological outcomes, identifying subset of samples with specific biological deviations. Generating additional ‘omics data including transcriptomic, metabolomic, lipidomic, and proteomics using both bulk and single cell/nuclei material alongside newer techniques such as spatial transcriptomics, would provide a more complete and nuanced understanding, revealing both population‐level and cellular heterogeneity. In addition to expanding the genetic analysis, the inclusion of additional epigenetic intermediates should be explored. Currently two small BDR studies have indicated that there is a general decrease in 5‐hydroxymethylcytosine, an intermediate methylation state, in AD compared with controls in the entorhinal cortex and cerebellum.^92^Subsequent analysis observed that the 5‐hydroxymethylcytosine modification demonstrates a tissue‐specific pattern across genomic regions,^93^highlighting the need for broader investigation of diverse genome modifications.

To facilitate this development of data‐driven hypotheses, the “BDR Collaborative” was established. The group encourages anyone using BDR samples to share their research, ideas, and expertise to start discussions and drive the generation of new innovative questions of this cohort, maximizing utility of the BDR resource via collaboration. The Collaborative currently comprises 40 members with a diversity of scientific expertise across multiple UK institutions, Europe and the United States: promoting and increasing the use of this crucial resource.

Initial efforts of the Collaborative are focused on aligning BDR data and results on matched samples, identifying consistent findings and parallel conclusions/hypotheses to follow‐up, incorporating interdisciplinary approaches. Combination of *post‐mortem* brain data with the matched longitudinal blood samples, allows a greater propensity for identifying blood‐based biomarkers, to aid in diagnosis and prediction of disease pathology. A fully integrated approach combined with machine‐learning promises to create data‐driven hypotheses for disease mechanisms, therapeutic targets, and precision medicine strategies.

Moving forward, large collaborative efforts supported by substantial funding are required to fully utilize these resources and providing freely available data for intersection will be key to advancing understanding of aging and dementia. This will aid in the identification of biomarkers, for more accurate disease diagnosis and potential early warning triggers for therapeutic interventions. Improved knowledge of disease aetiology and progression will also enable the discovery of novel drug targets and the development of tailored treatments, allowing a more proactive response to neurodegeneration.

Some might view the use of human *post‐mortem* tissue as limiting due its inability to fully disentangle mechanisms and distinguishing cause from consequence. Yet human *post‐mortem* brain tissue remains the cornerstone of biomedical research, offering unparalleled disease accuracy and relevance. The extensive use of BDR clearly demonstrates the research appetite for *post‐mortem* tissue and the critical importance of continued support for well‐characterized longitudinal projects.

## AUTHOR CONTRIBUTIONS

All authors contributed to the study conception and design. Relevant sections of the manuscript were written by named authors and drafts of the manuscript were compiled and edited by Keeley Brookes. All authors commented on previous versions of the manuscript. All authors read and approved the final manuscript.

## CONFLICT OF INTEREST STATEMENT

The authors declare no conflicts of interest. Author disclosures are available in the Supporting Information.

## Supporting information



Supporting Information

Supporting Information

## FULL LIST OF BDR COLLABORATIVE MEMBERS

### 1

Alan Thomas; Alexandra Stolzing; Andrew Robinson; Angela Hodges; Ben Middlehurst; Carolyn Sloan; Claire Troakes; Debbie Lett; Diego Gomez‐Nicola; Emma Anderson; Emma Dempster; Emma Kidd; Erin H Norris; Gayle Doherty; Henry Houlden; Iliya Lefterov; Irundika Dias; Jaleel Miyan; James Perry; Jerome Swinny; Jill Madine; Johanna Jackson; John Quinn; Jon Mill; Keeley Brookes; Laura Palmer; Laura Parkkinen; Lauren Walker; Lindsey Sinclair; Luc Zimmer; Lucy Devendra; Michael Harte; Nicky Barnett; Patrick Gavin Kehoe; Petronella Kettunen; Rebecca Sims; Richard Cain; Sara Rodrigues; Scott Miners; Wendy Noble.
