# Supplementary material for: Brains for dementia research at 18: Commitment to impact the value of human post‐mortem tissue
Source: Alzheimers Dement. 2026 Oct 26;22(10):e71956. doi: 10.1002/alz.71956 (PMC13710671; doi:10.1002/alz.71956)
Supplement: Supplementary file 2 [file ALZ-22-e71956-s002.docx]

**Supplemental Material:**

**Recruitment of Participants:**

| Year | Bristol | Cardiff | KCL | Manchester | Newcastle | Oxford |
| --- | --- | --- | --- | --- | --- | --- |
| 2009 | **0** | **65** | **140** | **85** | **72** | **71** |
| 2010 | **0** | **54** | **114** | **102** | **56** | **98** |
| 2011 | **63** | **27** | **38** | **34** | **74** | **84** |
| 2012 | **162** | **59** | **111** | **95** | **21** | **137** |
| 2013 | **190** | **163** | **123** | **104** | **70** | **130** |
| 2014 | **33** | **66** | **57** | **55** | **77** | **40** |
| 2015 | **22** | **30** | **7** | **52** | **64** | **35** |
| 2016 | **10** | **12** | **10** | **4** | **6** | **45** |
| 2017 | **5** | **11** | **2** | **3** | **8** | **7** |
| 2018 | **3** | **6** | **0** | **8** | **2** | **0** |
| TOTAL | **485** | **493** | **602** | **542** | **450** | **647** |

Table S1: Breakdown of participants recruited to the BDR project by centres within the recruitment period, totalling 3219 potential brain donations for research purposes.

**Frozen Post-mortem Brain Tissue:** At the point of donation, the brain is cut into half to separate the two hemispheres including the hindbrain. One of the hemispheres and half hindbrain are frozen. Following the separation of the brainstem and cerebellum from the cerebrum, the brainstem and cerebellum are sub-divided into the midbrain, pons, medulla and cerebellum. 1cm thick brain slices are taken from the cerebrum coronally, at least 8 regions dissected as small frozen blocks (cerebellum, frontal (BA9/46), cingulate, putamen, caudate nucleus, temporal (BA21/BA22), parietal (BA40) and calcarine) before all tissue is rapidly frozen and stored at approx. -80°C.

**Formalin-fixed Paraffin-embedded Tissue:**

A brain hemisphere and half hindbrain are fixed in 10% buffered formalin before being sampled and processed into FFPE blocks as per the table below.

| **Block number** | **Brain area** |
| --- | --- |
| 1 | Middle frontal gyrus (BA8/9) |
| 2 | Superior and middle temporal gyri (BA21) |
| 3 | Anterior hippocampus and parahippocampal gyrus |
| 4 | Superior parietal gyrus (BA40) |
| 5 | Mid brain at the level of the third cranial nerve |
| 6 | Superior frontal gyrus (inc. cingulate gyrus) (BA24 and 32) |
| 7 | Occipital cortex to include the calcarine sulcus |
| 8 | Striatum at the level of anterior commissure (inc. nucleus basalis of meynert and the hypothalamus). |
| 9 | Amygdala and the anterior entorhinal cortex. |
| 10 | Thalamus and subthalamic nucleus at level of lateral genucleate body. |
| 11 | Pons (rostral part) through midpoint of locus coeruleus |
| 12 | Medulla through the maximum diameter of inferior olive |
| 13 | Cerebellum through the superior cerebellar peduncle (inc. dentate nucleus). |
| 14 | Deep white matter of frontal lobe |
| 15 | Deep white matter of occipital lobe |
| 39 | Posterior hippocampus and parahippocampal gyrus (BA28,36) |

Table S2: FFPE blocks brain regions.

**Pathology Detection:**

| Pathology | Antibody | Source |
| --- | --- | --- |
| Tau | MAB MO AntiH PHF Tau AT8 (Mouse)  Phospho-Tau (Ser202, Thr205) Monoclonal Antibody (AT8) (Mouse) | Launch Diagnostics (90206)  ThermoFisher Scientific (MN1020) |
| 3R Tau | Anti-Tau (3-repeat isoform RD3) Antibody, clone 8E6/C11 (Mouse) | Merck Life Science UK Limited  (05-803) |
| 4R Tau | Anti-Tau (4-repeat isoform RD4) Antibody, clone 1E1/A6 (Mouse) | Merck Life Science UK Limited  (05-804) |
| Amyloid-β | Anti-β-Amyloid, 17-24 Antibody, clone 4G8 (Mouse)  Purified anti-β-Amyloid, 1-16 Antibody (Mouse) (Previously Covance catalog# SIG-39320) | BioLegend UK Ltd; (800708)  BioLegend UK Ltd: (803003) |
| α-Synuclein | 42/α-Synuclein (Mouse)  Alpha-Synuclein, clone KM51 (Mouse) | Becton Dickinson UK Ltd  (610787)  Leica Biosystems (NCL-L-ASYN) |
| TDP-43 | TDP-43 Polyclonal antibody (Rabbit)  Anti phospho TDP-43 (pS409/410) polyclonal antibody [replaces CAC-TIP-PTD-P01] (Rabbit) | Proteintech  (10782-2-AP)  2bScientific (CAC-TIP-PTD-P07) |
| P62 | 3/P62 LCK LIGAND (Mouse) | BD Biosciences (610832) |

Table S3: Antibodies used to detect pathology of relevance to dementia in BDR donated brain tissue, alongside dilutions utilised and manufacturer used across BDR Brain Banks for staging of pathology.

**Fibroblast Store:**

|  | Number of fibroblasts Total=164 |
| --- | --- |
| Awaiting PM report | **50** |
| Control | **42** |
| AD | **37** |
| Dementia with Lewy Bodies | **8** |
| Vascular Dementia | **6** |
| MCI | **7** |
| Intermediate Pathology | **6** |
| PART | **4** |
| PSP | **4** |

Table S4: Breakdown of fibroblast cells available from BDR participants by pathological diagnosis.

**References for staging:**

•	Montine TJ, Phelps CH, Beach TG, Bigio EH, Cairns NJ, Dickson DW, Duyckaerts C, Frosch MP, Masliah E, Mirra SS, Nelson PT, Schneider JA, Thal DR, Trojanowski JQ, Vinters HV, Hyman BT; National Institute on Aging; Alzheimer’s Association (2012) National Institute on Aging-Alzheimer’s Association guidelines for the neuropathologic assessment of Alzheimer’s disease: a practical approach. Acta Neuropathol 123, 1-11.

•	McKeith IG, Galasko D, Kosaka K, Perry EK, Dickson DW, Hansen LA, Salmon DP, Lowe J, Mirra SS, Byrne EJ, Lennox G, Quinn NP, Edwardson JA, Ince PG, Bergeron C, Burns A, Miller BL, Lovestone S, Collerton D, Jansen EN, Ballard C, de Vos RA, Wilcock GK, Jellinger KA, Perry RH (1996) Consensus guidelines for the clinical and pathologic diagnosis of dementia with Lewy bodies (DLB): report of the consortium on DLB international workshop. Neurology 47, 1113-1124.

•	McKeith IG, Dickson DW, Lowe J, Emre M, O'Brien JT, Feldman H, Cummings J, Duda JE, Lippa C, Perry EK, Aarsland D, Arai H, Ballard CG, Boeve B, Burn DJ, Costa D, Del Ser T, Dubois B, Galasko D, Gauthier S, Goetz CG, Gomez-Tortosa E, Halliday G, Hansen LA, Hardy J, Iwatsubo T, Kalaria RN, Kaufer D, Kenny RA, Korczyn A, Kosaka K, Lee VM, Lees A, Litvan I, Londos E, Lopez OL, Minoshima S, Mizuno Y, Molina JA, Mukaetova-Ladinska EB, Pasquier F, Perry RH, Schulz JB, Trojanowski JQ, Yamada M; Consortium on DLB (2005) Diagnosis and management of dementia with Lewy bodies: third report of the DLB Consortium. Neurology 65, 1863-1872.

•	McKeith IG, Boeve BF, Dickson DW, Halliday G, Taylor JP, Weintraub D, Aarsland D, Galvin J, Attems J, Ballard CG, Bayston A, Beach TG, Blanc F, Bohnen N, Bonanni L, Bras J, Brundin P, Burn D, Chen-Plotkin A, Duda JE, El-Agnaf O, Feldman H, Ferman TJ, Ffytche D, Fujishiro H, Galasko D, Goldman JG, Gomperts SN, Graff-Radford NR, Honig LS, Iranzo A, Kantarci K, Kaufer D, Kukull W, Lee VMY, Leverenz JB, Lewis S, Lippa C, Lunde A, Masellis M, Masliah E, McLean P, Mollenhauer B, Montine TJ, Moreno E, Mori E, Murray M, O'Brien JT, Orimo S, Postuma RB, Ramaswamy S, Ross OA, Salmon DP, Singleton A, Taylor A, Thomas A, Tiraboschi P, Toledo JB, Trojanowski JQ, Tsuang D, Walker Z, Yamada M, Kosaka K (2017) Diagnosis and management of dementia with Lewy bodies: Fourth consensus report of the DLB Consortium. Neurology 89, 88-100.

•	Attems J, Toledo JB, Walker L, Gelpi E, Gentleman S, Halliday G, Hortobagyi T, Jellinger K, Kovacs GG, Lee EB, Love S, McAleese KE, Nelson PT, Neumann M, Parkkinen L, Polvikoski T, Sikorska B, Smith C, Grinberg LT, Thal DR, Trojanowski JQ, McKeith IG. Neuropathological consensus criteria for the evaluation of Lewy pathology in post-mortem brains: a multi-centre study. Acta Neuropathol. 2021 Feb;141(2):159-172. doi: 10.1007/s00401-020-02255-2. Epub 2021 Jan 5. PMID: 33399945; PMCID: PMC7847437.

•	Mackenzie IR, Neumann M, Bigio EH, Cairns NJ, Alafuzoff I, Kril J, Kovacs GG, Ghetti B, Halliday G, Holm IE, Ince PG, Kamphorst W, Revesz T, Rozemuller AJ, Kumar-Singh S, Akiyama H, Baborie A, Spina S, Dickson DW, Trojanowski JQ, Mann DM (2009) Nomenclature for neuropathologic subtypes of frontotemporal lobar degeneration: consensus recommendations. Acta Neuropathol 117, 15-18.

•	Mackenzie IR, Neumann M, Baborie A, Sampathu DM, Du Plessis D, Jaros E, Perry RH, Trojanowski JQ, Mann DM, Lee VM (2011) A harmonized classification system for FTLD-TDP pathology. Acta Neuropathol 122, 111-113.

•	Dickson DW, Bergeron C, Chin SS, Duyckaerts C, Horoupian D, Ikeda K, Jellinger K, Lantos PL, Lippa CF, Mirra SS, Tabaton M, Vonsattel JP, Wakabayashi K, Litvan I; Office of Rare Diseases of the National Institutes of Health (2002) Office of Rare Diseases neuropathologic criteria for corticobasal degeneration. J Neuropathol Exp Neurol 61, 935-946.

•	Hauw JJ, Daniel SE, Dickson D, Horoupian DS, Jellinger K, Lantos PL, McKee A, Tabaton M, Litvan I (1994) Preliminary NINDS neuropathologic criteria for Steele-Richardson-Olszewski syndrome (progressive supranuclear palsy) Neurology 44, 2015-2019.

•	Trojanowski JQ, Revesz T; Neuropathology Working Group on MSA (2007) Proposed neuropathological criteria for the post mortem diagnosis of multiple system atrophy. Neuropathol Appl Neurobiol 33, 615-620.

•	Braak H, Braak E (1998) Argyrophilic grain disease: frequency of occurrence in different age categories and neuropathological diagnostic criteria. J Neural Transm (Vienna) 105, 801-819.

•	Crary JF, Trojanowski JQ, Schneider JA, Abisambra JF, Abner EL, Alafuzoff I, Arnold SE, Attems J, Beach TG, Bigio EH, Cairns NJ, Dickson DW, Gearing M, Grinberg LT, Hof PR, Hyman BT, Jellinger K, Jicha GA, Kovacs GG, Knopman DS, Kofler J, Kukull WA, Mackenzie IR, Masliah E, McKee A, Montine TJ, Murray ME, Neltner JH, Santa-Maria I, Seeley WW, Serrano-Pozo A, Shelanski ML, Stein T, Takao M, Thal DR, Toledo JB, Troncoso JC, Vonsattel JP, White CL 3rd, Wisniewski T, Woltjer RL, Yamada M, Nelson PT (2014) Primary age-related tauopathy (PART): a common pathology associated with human aging. Acta Neuropathol 128, 755-766.

•	Kovacs GG, Ferrer I, Grinberg LT, Alafuzoff I, Attems J, Budka H, Cairns NJ, Crary JF, Duyckaerts C, Ghetti B, Halliday GM, Ironside JW, Love S, Mackenzie IR, Munoz DG, Murray ME, Nelson PT, Takahashi H, Trojanowski JQ, Ansorge O, Arzberger T, Baborie A, Beach TG, Bieniek KF, Bigio EH, Bodi I, Dugger BN, Feany M, Gelpi E, Gentleman SM, Giaccone G, Hatanpaa KJ, Heale R, Hof PR, Hofer M, Hortobágyi T, Jellinger K, Jicha GA, Ince P, Kofler J, Kövari E, Kril JJ, Mann DM, Matej R, McKee AC, McLean C, Milenkovic I, Montine TJ, Murayama S, Lee EB, Rahimi J, Rodriguez RD, Rozemüller A, Schneider JA, Schultz C, Seeley W, Seilhean D, Smith C, Tagliavini F, Takao M, Thal DR, Toledo JB, Tolnay M, Troncoso JC, Vinters HV, Weis S, Wharton SB, White CL 3rd, Wisniewski T, Woulfe JM, Yamada M, Dickson DW (2016) Aging-related tau astrogliopathy (ARTAG): harmonized evaluation strategy. Acta Neuropathol 131, 87-102.

•	Nelson PT, Dickson DW, Trojanowski JQ, Jack CR, Boyle PA, Arfanakis K, Rademakers R, Alafuzoff I, Attems J, Brayne C, Coyle-Gilchrist ITS, Chui HC, Fardo DW, Flanagan ME, Halliday G, Hokkanen SRK, Hunter S, Jicha GA, Katsumata Y, Kawas CH, Keene CD, Kovacs GG, Kukull WA, Levey AI, Makkinejad N, Montine TJ, Murayama S, Murray ME, Nag S, Rissman RA, Seeley WW, Sperling RA, White CL 3rd, Yu L, Schneider JA (2019) Limbic-predominant age-related TDP-43 encephalopathy (LATE): consensus working group report. Brain 142,(6) 1503-1527.

•	Skrobot OA, Attems J, Esiri M, Hortobágyi T, Ironside JW, Kalaria RN, King A, Lammie GA, Mann D, Neal J, Ben-Shlomo Y, Kehoe PG, Love S (2016) Vascular cognitive impairment neuropathology guidelines (VCING): the contribution of cerebrovascular pathology to cognitive impairment. Brain 139, 2957-2969.
